# Machine Learning Techniques for the Detection of Shockable Rhythms in Automated External Defibrillators

**DOI:** 10.1371/journal.pone.0159654

**Published:** 2016-07-21

**Authors:** Carlos Figuera, Unai Irusta, Eduardo Morgado, Elisabete Aramendi, Unai Ayala, Lars Wik, Jo Kramer-Johansen, Trygve Eftestøl, Felipe Alonso-Atienza

**Affiliations:** 1 Department of Telecommunication Engineering, Universidad Rey Juan Carlos, Madrid, Spain; 2 Department of Communication Engineering, University of the Basque Country UPV/EHU, Bilbao, Spain; 3 Electronics and Computing Department, University of Mondragon, Mondragon, Spain; 4 Norwegian National Advisory Unit on Prehospital Emergency Medicine (NAKOS), Oslo University Hospital and University of Oslo, Oslo, Norway; 5 Department of Electrical Engineering and Computer Science, University of Stavanger, Stavanger, Norway; University of Minnesota, UNITED STATES

## Abstract

Early recognition of ventricular fibrillation (VF) and electrical therapy are key for the survival of out-of-hospital cardiac arrest (OHCA) patients treated with automated external defibrillators (AED). AED algorithms for VF-detection are customarily assessed using Holter recordings from public electrocardiogram (ECG) databases, which may be different from the ECG seen during OHCA events. This study evaluates VF-detection using data from both OHCA patients and public Holter recordings. ECG-segments of 4-s and 8-s duration were analyzed. For each segment 30 features were computed and fed to state of the art machine learning (ML) algorithms. ML-algorithms with built-in feature selection capabilities were used to determine the optimal feature subsets for both databases. Patient-wise bootstrap techniques were used to evaluate algorithm performance in terms of sensitivity (Se), specificity (Sp) and balanced error rate (BER). Performance was significantly better for public data with a mean Se of 96.6%, Sp of 98.8% and BER 2.2% compared to a mean Se of 94.7%, Sp of 96.5% and BER 4.4% for OHCA data. OHCA data required two times more features than the data from public databases for an accurate detection (6 vs 3). No significant differences in performance were found for different segment lengths, the BER differences were below 0.5-points in all cases. Our results show that VF-detection is more challenging for OHCA data than for data from public databases, and that accurate VF-detection is possible with segments as short as 4-s.

## Introduction

Out-of-hospital cardiac arrest (OHCA) is a leading cause of death in the industrialized world, with an estimated annual incidence that varies between 52.5 (in Asia) and 111.9 (in Australia) per 100,000 person-years [1]. Lethal ventricular arrhythmias are one of the most frequent causes of OHCA. A defibrillation shock is the only effective way to treat lethal ventricular arrhythmias, and early defibrillation is one of the key factors in survival from OHCA [2]. In an out-of-hospital setting defibrillation shocks may be administered by lay-people before the arrival of the ambulance, using an automated external defibrillator (AED). AEDs include a shock advise algorithm (SAA) that analyzes the surface electrocardiogram (ECG), and delivers an electric shock if either rapid ventricular tachycardia (VT) or ventricular fibrillation (VF) are detected by the SAA.

**Fig 1:**
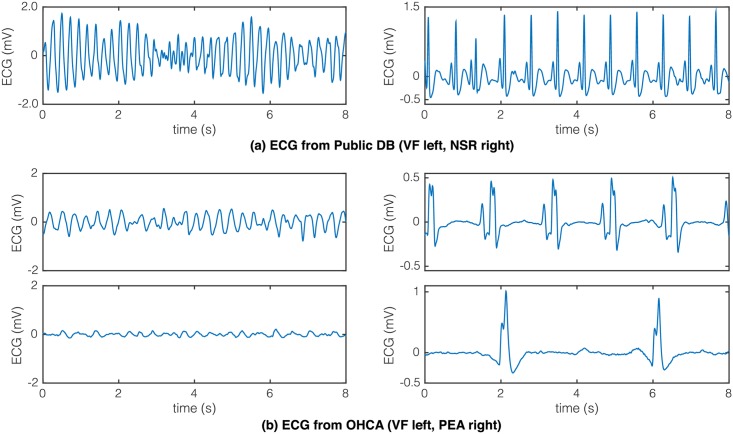
Examples of ECG found in public (top) and OHCA (bottom) data. The top-left segment corresponds to a VF from CUDB (record, cu05) right after VF onset, and presents large amplitude and a fibrillation frequency of 4.5 Hz. The bottom-left segments were recorded during OHCA 5–10 minutes after VF onset, and have smaller amplitudes and fibrillation frequencies (3.5 Hz and 2.5 Hz). The top-right segment corresponds to a NSR from cu05 right before VF onset. The bottom-right segments are examples of PEA in OHCA patients. Both cases show aberrant QRS complexes and low heart rates. The bottom example presents an extremely low heart-rate of 15 beats per minute.

The American Heart Association (AHA) defined the framework to test SAAs in AEDs [3]. The AHA recommends a sensitivity (Se) higher that 90% for shockable (Sh) rhythms, and a specificity (Sp) higher than 95% for nonshockable (NSh) rhythms, and above 99% in the case of normal sinus rhythms (NSR). The ECG segments used to test the SAA must be artefact-free and contain a single rhythm. During the last decades, a large number of features, methods and algorithms have been proposed to detect Sh rhythms within the AED setting [4–17]. Most of these studies are based on data from public databases, such as the MIT-BIH Arrhythmia Database (MITDB) [18], the MIT-BIH Malignant Ventricular Arrhythmia database (VFDB) [19], the Creighton University Ventricular Tachycardia database (CUDB) [20], and/or the AHA database (AHADB). Public databases contain a selection of long-term Holter ECG recordings. Thus, in general the onset of Sh events is clearly identified. VF records present a coarse amplitude and a high fibrillation frequency. NSh rhythms often correspond to NSR with narrow QRS complexes and normal rates. These data may be very different from the ECG recorded during OHCA, as shown in Fig 1. During OHCA, ECG signals are recorded by defibrillators normally 5–10 minutes after the onset of the cardiac arrest event. VF then presents smaller amplitudes and fibrillation frequencies [21], and the most frequent NSh rhythms are asystole (AS) and pulseless electrical activity (PEA). PEA often presents a bradyarrhythmic ECG with aberrant QRS complexes.

Within the AHA framework, this study explores the differences in the detection of Sh rhythms when public or OHCA data are used to optimize the algorithms. Following a machine learning approach as in [16, 17], we used a combination of 30 previously defined ECG features [4–17]. We then fed the values of the features to five state-of-the-art machine learning classifiers. The classifiers were selected to allow ranking of the features, which ultimately leads to a better insight into the relation between features and classification outcomes. All the routines, feature values, results and public data used for this study are available at http://www.tsc.urjc.es/~felipe.alonso/ohca_vs_public_dbs.html.

The paper is organized as follows. Materials and Methods presents the methodology including the ECG databases, the ECG features, the classifiers, and the feature selection procedure. Results analyzes the performance of the proposed algorithms. Finally discussion and conclusions are drawn in Discussion.

## Materials and Methods

### Overview of the procedure

**Fig 2:**
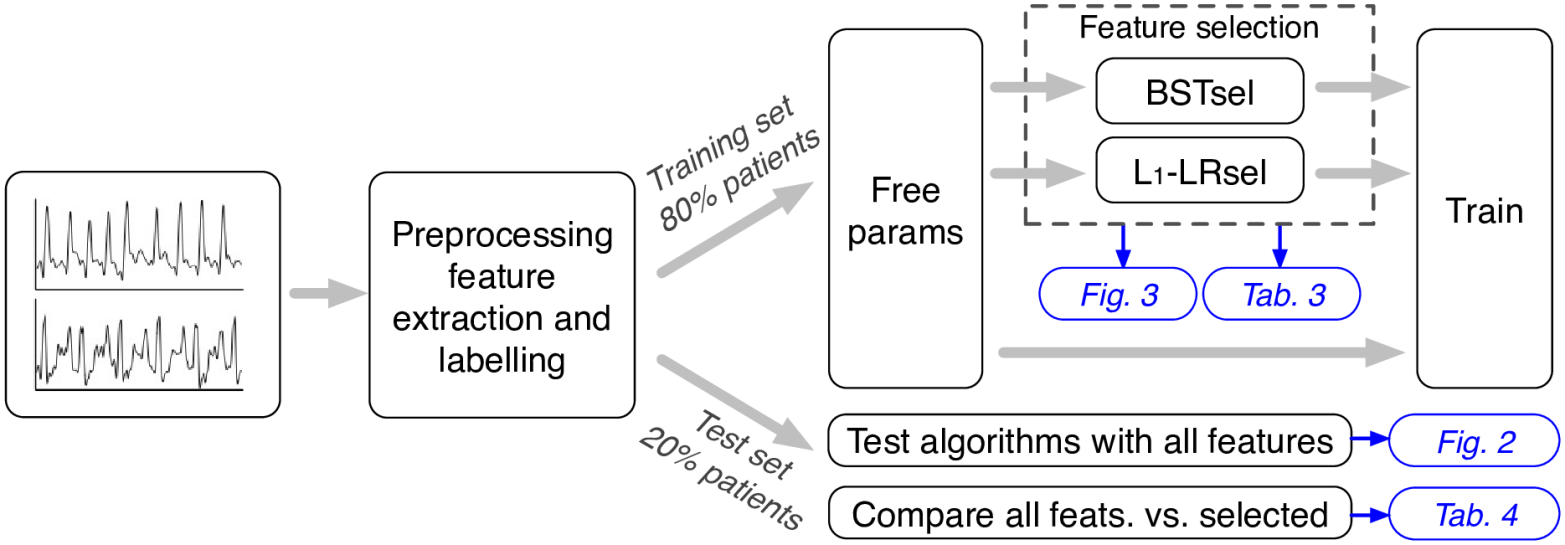
Overview of the test procedure. Blue boxes specify the figures and tables where the results corresponding to each procedure can be found in the manuscript.

This section provides an overview of the procedures described in the materials and methods, which are visually summarized in Fig 2. The process was done independently for public and OHCA data. First, ECG signals were preprocessed, labelled and divided into consecutive non-overlapping segments. For each segment thirty features were computed. Then data was split in training and test sets randomly, by allocating 80/20% of the patients to the training and test sets, respectively. Three steps were followed using the data in the training subset: (i) tuning the parameters of the classification algorithms (free parameters); (ii) feature selection using bootstrap resampling; and (iii) training the algorithms. Two different methods were used for feature selection (BSTsel and *L*_1_-LRsel). Finally, the selected features and the optimized algorithms were used on the test set to report the final results, and to compare feature selection against using all features.

### ECG collection

We used two databases of ECG recordings: a database of rhythms covered in the AHA recommendations built from public repositories, and a database of OHCA rhythms.

#### Public database

We included the complete set of records of the VFDB and the CUDB, and ten episodes of the AHADB series 1 (8201–8210). The VFDB contains 22 30-min long Holter record files with two channels per file. The CUDB contains 35 8-min long records from patients who experienced sustained episodes of lethal ventricular arrhythmias. Finally, the AHADB records are 35-min long with two channels, and contain annotated rhythms with lethal ventricular arrhythmias. In VFDB and AHADB only the first channel was included to avoid redundancy in the algorithms’ learning process. The sample rate of all databases was 250 Hz.

The original rhythm annotations of CUDB and VFDB were revised by consensus among two experienced biomedical engineers. Re-annotation comprised the relabelling of noise and device saturation intervals, the labelling of low peak-to-peak amplitude VF (under 200 μV) as fine VF [3], and of intervals with low rates (below 12 bpm) and/or very low peak-to-peak amplitudes (< 100 μV) as AS. Fine VF and AS labels were introduced to annotate the data in accordance with the AHA framework and the standard criteria used to annotate cardiac arrest rhythms [3, 22, 23].

#### OHCA database

The OHCA database was obtained from a multicentre cardiac arrest study conducted to evaluate cardiopulmonary resuscitation quality [24, 25]. Rhythm annotations on the data were done by clinical experts using five classes: VF, VT, PEA, pulse generating rhythms (PR) and AS [24]. Artifact-free ECG segments of 10-s duration and with a unique rhythm were extracted. The surface ECG was acquired using a modified Laerdal HeartStart 4000 defibrillator, at a sampling rate of 500 Hz and 16 bits for a resolution of 1.031 *μ*V per least significant bit. The ECG was resampled for this study to 250 Hz.

#### Preprocessing

ECG signals from all databases were preprocessed using the filtering process proposed in [11]: 1) mean subtraction; 2) five-order moving average filter; 3) high-pass filter with *f*_*c*_ = 1 Hz (drift suppression); and 4) low-pass Butterworth filter with *f*_*c*_ = 30 Hz. The 1–30 Hz is a typical monitor bandwidth used in AEDs [22, 26].

#### Data labelling

The final datasets were constructed and labelled following the AHA framework. Data segments in which the ECG did not conform to the specifications of the AHA framework, and to the standard practices used in VF-detection algorithms were excluded from the analyses [13, 17]. First, noise and low-quality ECG segments (artifacts) were excluded [3]. Then, ECG signals were divided into non-overlapping segments of 4-s and 8-s duration, and segments with rhythm transitions were excluded [3]. Intermediate rhythms such as slow VT (rate under 150 bpm) and fine VF were excluded [3, 13]. The benefits of defibrillation are unclear for these rhythms [3], and therefore they cannot be unequivocally classified as Sh or NSh. Finally, following standard practice in VF-detection algorithms rhythms with minimal electrical activity, such as AS, were also excluded [13, 17]. In SAAs asystole is customarily identified before the Sh/NSh decision using simple algorithms based on the amplitude/power of the ECG segment [27, 28].

The final segment datasets grouped by databases are shown in Table 1. Based on the original annotations, segments were labelled as Sh or NSh. Sh rhythms include VF, VT and ventricular flutter. NSh rhythms include NSR and arrhythmias like supraventricular tachycardia, atrial fibrillation, heart blocks or ectopic ventricular activity from public databases; and the PEA/PR rhythms from the OHCA database.

**Table 1:** Description of the datasets used for classification.

		4-s segments		8-s segments	
Database	patients	Sh	NSh	Sh	NSh
**Public**	**67**	**3578**	**14495**	**1696**	**7086**
vfdb	22	1586	7761	746	3780
cudb	35	716	2986	323	1446
ahadb	10	1276	3748	627	1860
**OHCA**	**260**	**680**	**1294**	**340**	**647**

The Sh category includes VF, VT and ventricular flutter. The NSh category includes: NSR, supraventricular tachycardia, sinus bradycardia, atrial fibrillation, ventricular bigeminy, ectopic ventricular activity, blocks, ventricular escapes, nodal and paced rhythms from public databases, and PEA/PR from the OHCA database.

### ECG features

For each segment a set of 30 VF-detection features was computed. A detailed description of the features can be found in the original papers [4–17]. In brief, these features quantify a distinctive VF characteristic and can be grossly grouped into (the nomenclature of the features follows that of the original papers):

- *Temporal features* to characterize the amplitude, slope, sample distribution or heart rate of the rhythm. The features include: threshold crossing interval (TCI) [7]; threshold crossing sample count (TCSC) [15]; standard exponential (Exp) [11]; modified exponential (Expmod) [11]; mean absolute value (MAV) [14]; count1, count2 and count3 [10]; x1, and x2 [23]; bCP [27].
- *Spectral features* to quantify spectral concentration, normalized spectral moments or the relative power content in different frequency bands. The features include: VF filter (vFleak) [4]; M, A1, A2, and A3 [6]; x3, x4, and x5 [23]; bWT [27].
- *Time-frequency features*. The Li feature [29] based on the wavelet analysis of the ECG.
- *Complexity features*. The most representative measures of the complexity of the ECG, including: complexity measure (CM) [9]; covariance (CVbin), area (abin), frequency (Frqbin), and Kurtosis (Kurt) of a binary signal extracted from the ECG [13]; and the phase space reconstruction (PSR) [12]; Hilbert transform (HILB) [12]; Sample entropy (SamEn) [30].

Features count1, count2 and count3 were normalized to the window size, and Kurt, M, A1, x1, x3, x5 and count3, were transformed using nonlinear operations to avoid skewed histograms.

#### Dataset for classification

The parametrization of the ECG signal segments resulted in a dataset of binary labeled data **Z** = {(**x**_1_, *y*_1_), …, (**x**_*N*_, *y_N_*)}, where $x_{i}\in R^{K}$, *K* = 30 (number of features), *N* = 20047/9769 (number of 4/8-s segments), and labels *y*_*i*_ ∈ {Sh: +1, NSh: –1}. During the classification process, features were standardized to zero mean and unit variance using the data in the training set.

### Classification algorithms

This section presents an abridged description of the five classifiers selected for this study, for further details consult [31].

#### *L*_1_ regularized logistic regression (*L*_1_-LR)

This is an extension of the classical logistic regression. In *L*_1_-LR the **w**^*T*^ = [*w*_1_, *w*_2_, …, *w*_*K*_] regression coefficients are obtained as follows:

$$\max_{w_{0},w}\left\{\sum_{i=1}^{N}\left[\left(1-y_{i}\right)\left(w_{0}+w^{T}x_{i}\right)\right]+\ln\left(1+e^{-(w_{0}+w^{T}x_{i})}\right)-\lambda \sum_{k=1}^{K}\left|w_{k}\right|\right\}.$$ (1)

The *L*_1_-LR method yields a sparse vector **w** (few nonzero coefficients), that can be used as a feature selection method. The sparsity of **w** is controlled by the regularization parameter *λ*. High values of *λ* would force all coefficients to be zero, while low values of *λ* result in coefficient values greater than zero.

To minimize classification errors *λ* is set a priori as a balance between algorithm complexity and accuracy. We used a 10-fold cross validation to determine *λ*.

#### Ensemble methods

These are general procedures to combine outcomes of a set of classifiers (classification trees) to improve prediction performance. Three approaches are explored in this study:

- *Bagging (BAG) and Random Forests (RF)*. Bagging (BAG) constructs *B* decision trees from *B* bootstrap samples of the training database. The final decision is the majority vote of those *B* trees. Although *B* has to be set a priori, its value is not critical, sufficiently large values lead to good performance without overfitting [31]. Random Forest (RF) is a particular implementation of bagging for decision trees, in which only a random subset of *p* < *K* features are used in each of the *B* trees. This generates uncorrelated trees, reducing the variance of the classifier and improving its performance. The value of *p* is normally set to $p=\sqrt{K}$. Besides *B*, in BAG and RF the complexity of the trees has to be set a priori. For this purpose, we analyzed the out-of-bag missclassification error [31].
- *Boosting (BST)* combines many *weak* classifiers to improve accuracy. For *M* boosting iterations a sequence of *f*_*m*_(**x**) weak classifiers is constructed. At iteration *m* the observations misclassified by *f*_*m*−1_(**x**) have their weights increased, and those correctly classified have their weights decreased. So at the next iteration, *f*_*m*_(**x**) is forced to focus on samples that were difficult to classify in the previous iteration. The final classification is obtained by a weighted vote of the classifiers:
  $$y=\text{sign}\left(\sum_{m=1}^{M}\alpha_{m}f_{m}\left(x\right)\right).$$ (2)
  Choosing *f*_*m*_(**x**) to be decision trees three elements have to be set a priori: (i) the complexity of the trees; (ii) the reweighting strategy and the aggregation weights *α*_*m*_; and (iii) the number of iterations *M*. These three parameters were analyzed using 10-fold cross validation.

#### Support Vector Machine (SVM)

SVMs have been frequently used as binary classifiers [32]. In the dual formulation the SVM solves the following optimization problem:

$$\max_{\alpha_{i}}\left\{\sum_{i=1}^{N}\alpha_{i}-\frac{1}{2}\sum_{i,j=1}^{N}\alpha_{i}\alpha_{j}y_{i}y_{i}K\left(x_{i},x_{j}\right)\right\},s.t.\;0\leq \alpha_{i}\leq C\;\text{and}\;\sum_{i}\alpha_{i}y_{i}=0,\forall i,$$ (3)

where the coefficients *α*_*i*_ are non-zero only for *N*_*s*_ support vectors, *K*(**x**_*i*_, **x**_*j*_) is the kernel function and *C* the soft margin parameter. For this work we used gaussian kernel, *K*(**x**_*i*_, **x**_*j*_) = exp(−*γ*||**x**_*i*_ − **x**_*j*_||^2^). Once the support vectors are determined (optimal *α*_*i*_) the classifier output *y* for an input sample **x** is:

$$y=\text{sgn}\left(\sum_{i=1}^{N_{s}}\alpha_{i}y_{i}K\left(x_{i},x\right)+b\right),$$ (4)

where *b*, the intercept term, has a closed from expression in terms of *α*_*i*_, *y*_*i*_ and **x**_*i*_.

Model selection for this SVM involves estimating *C*, a tradeoff between training errors and complexity, and *γ*, the flexibility of the decision boundary. We used 10-fold cross validation to select *C* and *γ*.

### Performance metrics

The algorithms were assessed using performance metrics for binary diagnostic tests. In the paper we only report sensitivity and specificity, as specified by the AHA framework, and the Balanced Error Rate (BER):

$$\text{BER}=1-\frac{1}{2}\cdot \left(\text{Se}+\text{Sp}\right).$$ (5)

The BER is a balanced metric that equally weights errors in shockable (Se) and nonshockable (Sp) rhythm detection.

The statistical distribution of a given performance metric (*θ*) was estimated using patient-wise bootstrap resampling on the test set [31]. In total *B* = 500 resamples were used. Each resample was obtained by randomnly selecting *N* patients with replacement from the *N* patients in the set, which on average results in 2/3 of the patients being selected. In this way an empirical estimation of the distribution of the performance metrics was obtained [31]. To compare the performance of two algorithms paired bootstrap resampling was used, and the distribution of the difference in the metric (Δ*θ*) was estimated. No statistically significant differences in performance were assumed when the 95% confidence interval of Δ*θ* included the zero value.

Finally to avoid biases in the estimation of the performance metrics the bootstrap scheme was applied patient-wise so that patients included in the training bootstrap samples were not present in the test samples.

### Feature selection

One of the objectives of this work was to rank the ECG features in terms of detection performance, and to analyze the differences when data from public and OHCA databases were used. Selecting small feature subsets that preserve the overall accuracy of the Sh/NSh algorithms is very important in AED technology. AEDs are low-cost devices equipped with low-end microprocessors in a real-time application, therefore computational demands must be kept to a minimum by making the Sh/NSh algorithm as simple as possible. Moreover reducing the number of features will help to avoid overfitting. Two of the classification algorithms, BST and *L*_1_-LR, have an intrinsic feature selection capability since features can be ranked in the training phase in terms of importance (BST) [31], or in terms of the magnitude of the regression coefficients |*w*_*k*_| (*L*_1_-LR). In what follows feature selection based on these algorithms are denoted by BSTsel and *L*_1_-LRsel, respectively.

A patient-wise bootstrap procedure was run with *B* = 500 resamples. In each iteration, the resample is built by sampling with replacement the patients in the training subset. BST (for BSTsel) or *L*_1_-LR (for *L*_1_-LRsel) were trained with the selected samples and features were ranked as previously explained, and the least important feature was iteratively eliminated. For each feature subset size (*K* = 1, …, 30) the remaining samples were classified and the BER was computed. Then, using all bootstrap iterations, we selected the smallest number of features (*K*_*s*_) for which the mean BER was within one standard error of the lowest BER (subset selection threshold) [31]. Finally, we assigned a score to each feature according to the number of times the feature was selected through the bootstrap process, and the best *K*_*s*_ features were chosen as the optimal feature subset.

## Results

### Performance of individual parameters

The detection performance of each individual feature is reported in Table 2, ranked from top-left (best) to bottom-right (worse). The Se/Sp values for each feature and database were computed using a maximum likelihood classifier [31]. Hence, for the *i*-th feature with values ***x*_*i*_**, the optimal threshold is set to ***x***_*i*_: *f*(***x***_*i*_|*Sh*) = *f*(***x***_*i*_|*NSh*). Performance varies substantially across features, and no individual feature met AHA standards on all datasets. However, six features (bCP, x1, HILB, SamEn, bWT and PSR) had Se>90% and Sp>85% in all datasets, while other features (TCSC, MAV or VFleak) showed excellent performance only on public databases. Detection performance was better in public databases, with a median increase in Se/Sp with respect to the OHCA database of 3.7/5.4-points for 4-s segments, and 2.8/6.7-points for 8-s segments. Longer segment durations slightly increased the Se by 1.3-points, but only for the OHCA database.

**Table 2:** Performance analysis of single features for all datasets.

		Public (4-s and 8-s)		OHCA (4-s and 8-s)				Public (4-s and 8-s)		OHCA (4-s and 8-s)	
Feature		Se/Sp	Se/Sp	Se/Sp	Se/Sp	Feature		Se/Sp	Se/Sp	Se/Sp	Se/Sp
bCP	[27]	94.8/97.8	96.0/98.7	95.3/90.0	94.4/91.2	A2	[6, 11]	85.5/91.8	85.8/93.1	71.2/83.1	76.2/81.6
x1	[23]	95.6/96.3	95.8/96.5	93.8/91.1	94.7/89.5	TCI	[7, 11]	86.8/74.9	86.5/80.7	87.5/73.2	90.0/79.3
HILB	[12, 33]	96.5/93.3	95.8/93.7	93.8/88.7	92.4/87.3	x4	[23]	77.7/93.7	79.2/93.2	66.3/89.5	72.4/85.9
SamEn	[30]	94.9/91.6	96.6/92.1	91.3/89.9	91.5/91.2	Li	[29]	82.3/77.6	94.9/86.2	74.3/69.6	85.3/81.6
bWT	[27]	96.1/90.8	95.9/93.6	91.3/87.9	95.6/86.7	bW	[27]	90.6/88.5	93.5/88.9	80.1/60.1	86.2/55.8
PSR	[12, 33]	96.3/91.3	95.6/92.5	90.9/88.1	91.2/86.9	A3	[6, 11]	79.0/85.9	85.2/83.7	77.8/68.0	70.9/79.0
Count2	[10]	93.2/88.1	93.9/96.1	90.4/87.1	89.1/94.3	CM	[9, 11]	84.5/63.3	83.7/67.9	80.7/79.4	87.4/78.2
x2	[23]	95.0/95.0	92.8/96.0	90.4/87.1	87.9/85.6	M	[6, 11]	82.2/81.3	80.7/86.6	76.6/68.4	72.9/73.1
TCSC	[15]	95.3/91.0	97.1/92.4	91.5/81.4	92.4/83.0	Frqbin	[13, 17]	81.4/66.2	82.1/67.3	89.9/69.7	90.0/73.7
MAV	[14]	95.8/90.4	97.1/92.4	91.5/81.4	92.4/83.0	x5	[23]	86.6/78.9	89.5/78.9	87.4/41.3	88.2/40.3
Count3	[10]	90.3/85.5	94.6/90.6	86.5/84.1	92.1/87.6	CVbin	[13, 17]	91.8/47.2	89.0/48.8	88.7/55.3	90.9/56.0
vFleak	[4, 11]	94.4/93.1	96.2/92.7	78.7/87.4	83.2/85.2	abin	[13, 17]	92.3/46.6	90.6/47.1	89.0/54.9	90.9/56.0
Kurt	[13, 17]	96.3/87.4	96.9/87.8	91.2/76.3	87.6/80.1	x3	[23]	83.8/55.4	80.1/60.4	79.6/52.2	79.4/53.8
Count1	[10]	82.6/82.9	90.3/89.4	86.9/72.2	90.0/82.5	Exp	[11]	58.7/66.5	84.0/66.2	47.1/34.2	83.8/62.1
Expmod	[11]	86.5/78.1	90.0/77.9	87.1/83.7	90.6/81.9	A1	[6, 11]	14.1/92.9	14.2/93.6	15.4/79.0	14.4/77.7

Features are ranked (best on top-left, worst bottom-right) by average BER across all four datasets.

### Performance of classification algorithms

**Fig 3:**
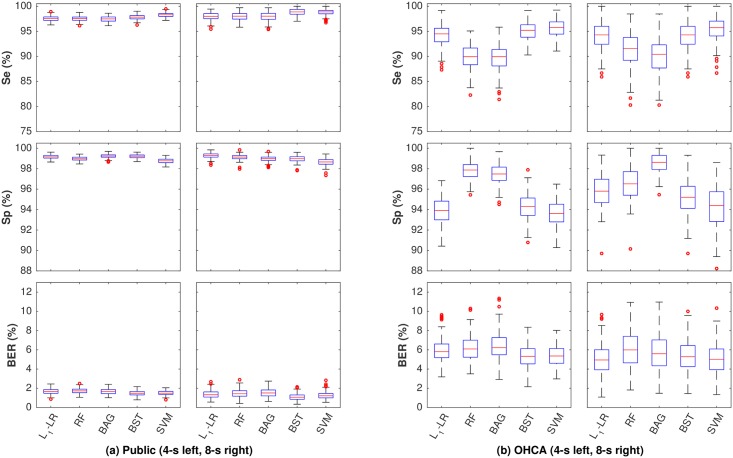
Box plots of the performance metrics for the five algorithms for the public databases (a) and the OHCA database (b). All features were included in the algorithms.

Fig 3 shows the box-plots of the performance metrics of the five classification algorithms when all features were included. The distributions of the metrics were obtained using patient-wise bootstrap (*B* = 500). In public databases, all algorithms met AHA performance recommendations with mean Se/Sp above 90%/95%, respectively. In the OHCA database the Sp of some was slightly below the 95% recommendation, with Se above 90% in all cases. All algorithms performed better for public databases than for OHCA databases, with a mean BER improvement above 4-points. There were no significant differences in performance within databases when different segment lengths were used (BER differences below 0.5-points). The best classifiers in terms of BER were SVM and BST, although *L*_1_-LR showed a similar performance for the OHCA data.

### Feature selection

**Fig 4:**
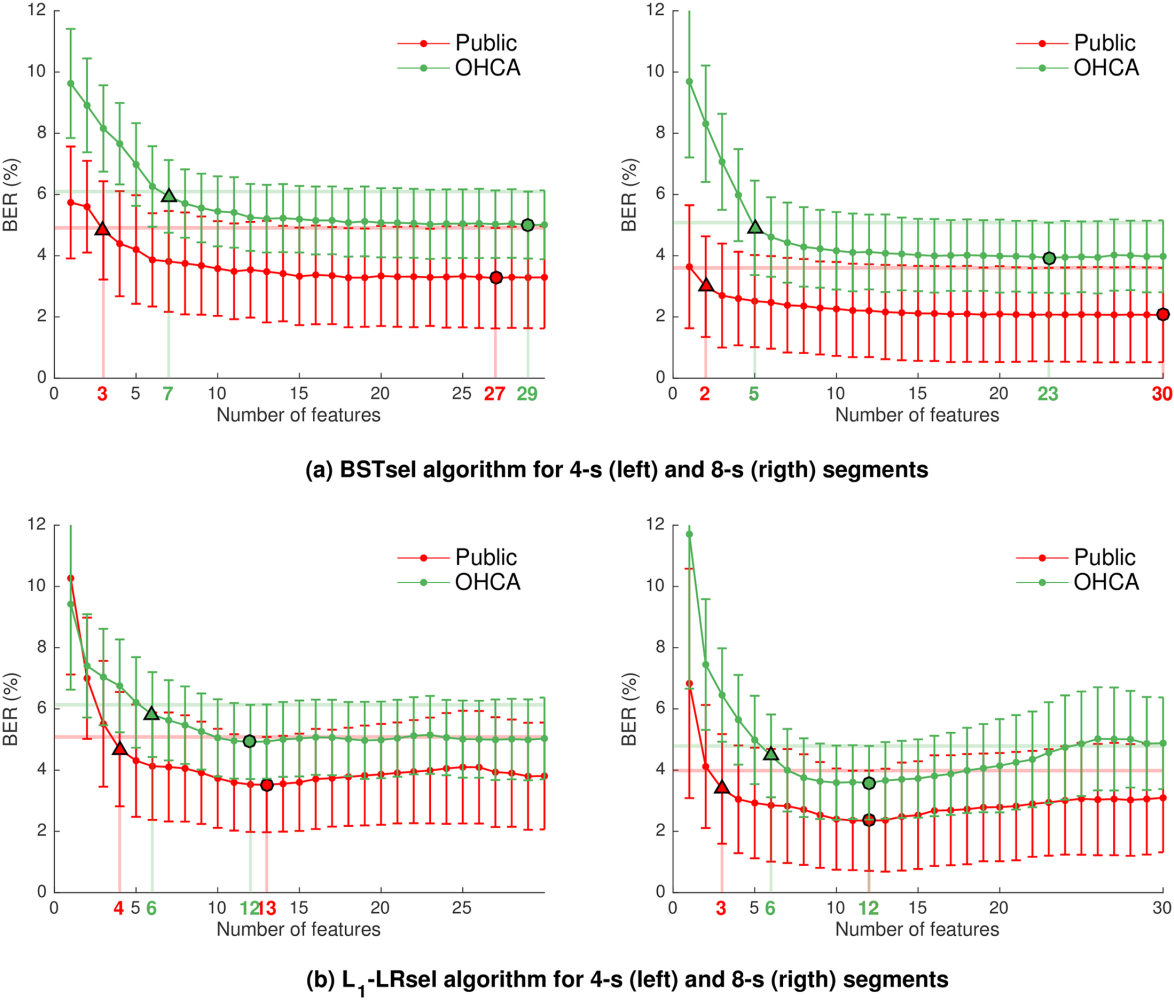
Feature selection with BSTsel (top) and the *L*_1_-LRsel (bottom) approaches. The results are shown for 4-s (left) and 8-s (right) segments for both public and OHCA databases. The mean BER is shown (with errorbars) for each subset size, and the horizontal line represents the subset selection threshold for the public (red) and OHCA (green) databases. The triangle and dot marks and their corresponding numbers represent the selected subset and the minimum BER subset, respectively.

Through feature selection we determined which features were important and which irrelevant for the Sh/NSh discrimination. The feature selection procedure is illustrated in Fig 4, for 4-s (left) and 8-s (right) segments and the BSTsel algorithm. The figure shows BER values (mean and standard-errors) for each subset size, and for both databases. The BER is smaller and changes less in public databases as more features are added, resulting in smaller optimal feature subsets.

Table 3 shows the features selected for the four datasets and the two feature selection methods. The results are consistent for a given dataset, but differ for public and OHCA databases. A specific set of features are selected in many cases, so they can be considered as robust features. These features were bCP and SamEn (selected in 6/8 cases), Li and vFleak (5/8 cases), bWT and x4 (4/8 cases). Table 4 shows the processor times required to compute the features when all features and optimal feature subsets are used. Processing times may vary depending on how feature calculation is implemented and on the processor used to compute the features. In our data, selecting optimal feature subsets reduced processing times to 1.3–22% of the time required to compute all features.

**Table 3:** Features selected with BSTsel and *L*_1_-LRsel ordered by decreasing relevance.

Method	Public-4s	Public-8s	OHCA-4s	OHCA-8s
BSTsel	bCP, vFleak, SamEn	bCP, vFleak	bCP, SamEn, bWT, x4, x1, Li, vFleak	bCP, Li, SamEn, x4, bWT,
*L*_1_-LRsel	vFleak, x2, Li, SamEn	vFleak, x2, bCP	bCP, x4, SamEn, bWT, Li, x1	Li, SamEn, x4, bWT, x1, A2

**Table 4:** Processing times (ms) to compute the features when all features and optimal feature subsets are computed.

Feature set	Public 4-s	Public 8-s	OHCA-4s	OHCA-8s
All features	3.73 (0.33)	5.38 (0.45)	3.81 (0.39)	5.77 (0.57)
Optimal, BSTsel	0.06 (0.01)	0.07 (0.01)	0.85 (0.08)	1.10 (0.08)
Optimal, *L*_1_-LRsel	0.46 (0.03)	0.24 (0.02)	0.83 (0.07)	1.58 (0.12)

Values are shown as mean and standard deviation in parenthesis. Calculations were made on a dedicated 2.8 GHz Intel Core i7 processor with 16 Gb of memory.

Finally, Table 5 shows the effect of feature selection on performance for 8-s segments in public and OHCA databases. The comparison is done in pairs for three cases: (i) all features (ALL); (ii) features selected with *L*_1_-LRsel; and (iii) features selected with BSTsel. No statistically significant differences were observed except in 6 of the 30 comparisons, in which the BER slightly increased when feature selection was applied.

**Table 5:** Decline in BER for the features selected using the *L*_1_-LR or BST algorithms.

	Public 8-s			OHCA 8-s		
Algorithm	ALL–BSTsel	ALL–*L*_1_-LRsel	BSTsel–*L*_1_-LRsel	ALL–BSTsel	ALL–*L*_1_-LRsel	BSTsel–*L*_1_-LRsel
*L*_1_—*LR*	-0.4 (-1.3, 0.3)	-0.3 (-0.9, 0.1)	0.1 (-0.8, 0.7)	-1.4 (-4.0, 0.2)	-1.4 (-4.3, 0.3)	0.0 (-2.2, 1.6)
RF	-0.2 (-1.1, 0.5)	0.1 (-0.5, 0.6)	0.4 (-0.3, 0.8)	-2.4 (-5.5, -0.1)*	-2.1 (-5.1, -0.3)	0.3 (-2.4, 2.4)
BAG	-0.6 (-1.4, -0.1)*	-0.6 (-1.5, -0.2)*	-0.1 (-0.2, 0.0)	-2.1 (-4.5, -0.7)*	-1.5 (-4.2, 0.5)	0.6 (-2.1, 2.7)
BST	-1.1 (-2.1, -0.5)*	-0.3 (-1.2, 0.3)	0.8 (0.1, 1.4)	-0.7 (-2.8, 0.7)	-2.1 (-5.3, 0.0)	-1.5 (-3.2, -0.3)*
SVM	0.4 (-0.4, 1.0)	0.3 (-0.4, 0.8)	-0.1 (-0.2, 0.0)	-0.7 (-2.7, 0.9)	-0.3 (-2.3, 1.2)	0.4 (-2.1, 2.0)

Values are shown as mean and 95% CI, and comparisons are made using a paired bootstrap procedure. Statistically significant differences are marked with an asterisk.

### ECG analysis

**Fig 5:**
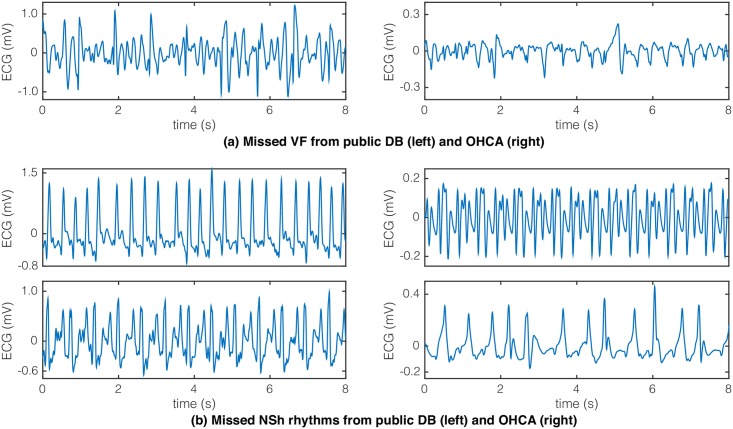
Examples of misclassified 8-s ECG samples from the public (left) and OHCA (right) databases. A VF is shown on top and two nonshockable rhythms below for both databases.

The misclassified ECG samples vary for the different features and algorithms. However, certain samples presented some salient characteristics that made them specially difficult to classify for any combination of features/algorithms. Some of those illustrative examples are shown in Fig 5, drawn both from the public and the OHCA databases.

One common source of misclassification errors in VF is the appearance of isolated QRS complexes, as shown in Fig 5 for the VF segment from the public databases. These QRS complexes have large slope values and/or important high frequency content that may result in feature values similar to those obtained for NSh rhythms. In the OHCA database VF frequently presents low amplitude and low fibrillation frequencies (below 2 Hz in the example), and may occasionally have isolated QRS complexes. Lower amplitudes and frequencies are more frequent in prolonged untreated VF, and these VF samples may be confused with low rate nonshockable ventricular rhythms. The Se for public databases is in average 5-points larger than for the OHCA database, so the prevalence of these VF in the OHCA database is much larger than in the public databases.

Fig 5 also shows examples of misclassified NSh rhythms from both databases. In the public databases the most frequent errors occur with fast supraventricular rhythms and rhythms with aberrant QRS complexes (conduction problems). In the OHCA cases most errors correspond to slower ventricular rhythms and rhythms with aberrant QRS complexes appearing during PEA. The Sp value is above 99% for public databases and around 97% for the OHCA database (see Fig 3), which indicates that borderline VF-PEA (or VF-NSh) cases are more frequent in OHCA than in the public databases.

## Discussion

This work is a comprehensive analysis of the detection of shockable rhythms based on the surface ECG, i.e. for use in AEDs and monitor-defibrillators. We used data from patients who suffered an OHCA, and were therefore untreated for minutes or only treated by chest compressions of variable quality, an scenario that reflects the real life AED use. The study includes a large set of 30 of the best known features described in the specialized literature [4–17], which were combined using state of the art machine learning algorithms. The past fifteen years have seen formidable advances in the field of shockable rhythm detection including: the development of new ECG features [10, 12, 15, 27], the introduction of comparative studies on feature performance [11, 15, 34, 35], and the recent systematic use of machine learning methods to efficiently combine ECG features [16, 17]. Most of these advances were based either on proprietary data [9, 27, 35, 36] or in partially described subsets of data from public ECG databases [11, 15–17, 34], which hinders the reproducibility of the results and further verifiable progress. Furthermore, only a few of these studies used data from OHCA patients [37–39]. So, for most of the features/algorithms performance on rhythms seen by an AED in the field has not been thoroughly assessed. This study advances the field by making all the code, feature values, results and public data available to serve as baseline for future developments and to allow complete reproducibility of the results (http://www.tsc.urjc.es/~felipe.alonso/ohca_vs_public_dbs.html). Moreover, the study analyzes and compares feature/algorithm performance for OHCA and ECGs from public databases.

In this study most features showed acceptable performance for public datasets. These datasets are ambulatory recordings (Holter records) from patients who only in some cases suffered cardiac arrest [19, 20]. In those cases, malignant ventricular arrhythmias were recorded at the onset of the cardiac event. Our good results for these datasets are coherent with previous comparative assessments conducted on fewer features [11, 34, 35], and are a natural consequence of the data originally used to develop the features, which in most studies either came from public databases [12, 13, 15] or from controlled clinical procedures [7, 9]. For OHCA rhythms the median BER per feature degraded significantly, with an increase of over 4.5-points. OHCA patients experience the arrest 5–8 min before the medical services arrive on scene [40]. As reference, in our data the mean response time was 7.3 min (SD 3.7 min). By then, the ECG rhythm may have deteriorated to rhythms very different from those observed at the onset of the arrest, or during induced clinical procedures. For instance, VF may transition from its initial electrical phase (0–4 min) into the circulatory (4–10 min) and sometimes into its metabolic phase (>10 min) [41]. Over time VF waveform amplitude and frequency decreases [42], and its complexity increases [43]. Organized nonshockable rhythms normally correspond to pulseless patients (PEA) [44] and are frequently narrow QRS tachycardias (pseudo-PEA) or bradycardic rhythms with conduction problems and aberrant QRS complexes (true-PEA) [45]. Borderline VF-PEA rhythms and rapid supraventricular rhythms are not rare in OHCA, but seldom occur in the public databases customarily used to develop VF-detection features. Therefore, ECG records from OHCA databases should be used to design new VF detection features and new SAAs for use in defibrillators. This could result in improved VF detection features and an increase in the sensitivity and specificity of SAAs.

Meeting AHA recommendations on OHCA data implies the efficient combination of features through machine learning techniques. Previous works on VF detection have introduced techniques like k-NN [46], linear discriminant analysis [47], decision trees [48], neural networks [49] or SVMs [16, 17], but applied to limited sets of 5–15 features and using data from public databases. By using a comprehensive set of ECG features combined in machine learning algorithms with built in feature selection capabilities, we were able to rank the features and identify the optimal feature subsets for the public and OHCA datasets. This is an intrinsic advantage of BST or *L*_1_-LR classifiers over SVMs, and leads to a better insight into the relation between features and classification outcomes. Our analysis shows that optimal feature subsets of 4–7 features are sufficient to preserve the accuracy of the Sh/NSh algorithms. Identifying these smaller feature subsets is very important in SAA design for AEDs, because of the limitations in computational power of the low-end microprocessors used in AED technology. In all our approaches, OHCA data required larger feature subsets and produced worse Se/Sp results, again stressing the inherent difficulties in OHCA rhythm classification. Our optimal feature subsets reveal the importance of a multi-domain approach that may include the analysis of the ECG’s: slope (bCP), time-domain baseline content (bWT), spectral characteristics (vFLeak, x4), time-frequency features (Li), and waveform complexity (SamEn).

Another salient feature of VF detection addressed in this study is the duration of the analysis segment. We found that the optimal feature subsets and the Se/Sp results were similar for 8-s and 4-s segments in both public and OHCA data. Most previous comparative assessments were done using 8 s segments [11, 15, 16, 34], although segment length varies across studies in ranges from 4 to 10 s [13, 17, 48, 50]. Shortening the duration of the AED’s rhythm analysis may contribute to the survival of the patient. The AED’s analysis interval is part of the pause in chest compressions before defibrillation (pre-shock pause), and an increase of 5-s in the pre-shock pause may decrease the chances of survival by as much as 18% [51]. Currently, AED analyses require segments longer than 6 s [52]. Our results confirm that it could be safely shortened to 4 s, in line with some recently published data [38, 39, 53].

In summary, this study provides a comprehensive review of VF-detection applied to defibrillators, introduces new machine learning algorithms with feature detection capabilities and identifies optimal feature subsets for Sh/NSh classification in both public and OHCA data. By making available all the code, feature values, results and public data to allow full reproducibility we hope to encourage and speed further developments in the field.

## Supporting Information

S1 Table BER for the five algorithms for each public database. Mean (standard deviation) of the BER obtained with a bootstrap resampling method for the three public databases and the five classification algorithms.
(PDF)
