# Supplementary material for: Machine Learning Techniques for the Detection of Shockable Rhythms in Automated External Defibrillators
Source: PLoS One. 2016 Jul 21;11(7):e0159654. doi: 10.1371/journal.pone.0159654 (PMC4956226; doi:10.1371/journal.pone.0159654)
Supplement: S1 Table — Mean (standard deviation) of the BER obtained with a bootstrap resampling method for the three public databases and the five classification algorithms. (PDF) [file pone.0159654.s001.pdf]

Comparative assessment of algorithm performance for the three public databases

Table S1 shows the mean and standard deviation of the BER for the three public databases and the five classification algorithms. The results are obtained by using a bootstrap resampling method over the test set for each database, with B = 500 resamples. BER for CUDB are higher than for the other two databases. SVM is the algorithm that shows the most robust behavior for the three databases. The weighted mean of these values is consistent with the values provided in the manuscript.

	**L_1-LR**	**RAN**	**BAG**	**BST**	**SVM**
**CUDB**	6.47(1.49)	4.73(1.19)	4.17(1.24)	4.00(1.21)	2.54(0.82)
**VFDB**	2.08(0.46)	1.56(0.39)	2.05(0.44)	1.29(0.37)	1.69(0.40)
**AHADB**	0.85(0.28)	0.72(0.29)	0.55(0.24)	0.91(0.28)	1.05(0.30)

Table S1. BER for the five algorithms for each public database, mean (standard deviation).
